# Immunotherapy Versus Chemo-Immunotherapy as First-Line Treatment in Metastatic Non-Small Cell Lung Cancer Patients with PD-L1 TPS ≥ 50%: A Real-World Retrospective Study

**DOI:** 10.3390/jcm15062406

**Published:** 2026-03-21

**Authors:** Maral Martin Mildanoglu, Mehmet Haluk Yucel, Ebru Engin Delipoyraz, Erdem Sunger, Hakan Ozcelik, Sena Fidan, Cihat Terzioglu, Harun Muglu, Jamshid Hamdard, Burcin Cakan Demirel, Yasin Kutlu, Ozgur Acikgoz, Mesut Seker, Ahmet Bilici

**Affiliations:** Department of Medical Oncology, Istanbul Medipol University, 34214 Istanbul, Turkey; mhalukyucel@gmail.com (M.H.Y.); drebruengin@gmail.com (E.E.D.); erdemsunger@gmail.com (E.S.); hknozcelikk@gmail.com (H.O.); senafidan03@gmail.com (S.F.); cihatterzioglu@gmail.com (C.T.); ahmetknower@yahoo.com (A.B.)

**Keywords:** NSCLC, PD-L1, immunotherapy, chemo-immunotherapy, prognostic

## Abstract

**Background**: Immune checkpoint inhibitors (ICIs) have become the standard first-line treatment for patients with metastatic non-small cell lung cancer (NSCLC) with high programmed death-ligand 1 (PD-L1) expression. However, the optimal selection between immunotherapy monotherapy and chemo-immunotherapy in patients with a PD-L1 tumor proportion score (TPS) ≥ 50% remains uncertain in routine clinical practice. **Methods**: We retrospectively reviewed patients with metastatic NSCLC and a PD-L1 TPS ≥ 50% who initiated first-line treatment with pembrolizumab monotherapy or pembrolizumab combined with platinum-based chemotherapy at the Istanbul Medipol University Department of Medical Oncology between July 2017 and December 2024. Survival outcomes, including progression-free survival (PFS) and overall survival (OS), were evaluated and compared according to PD-L1 expression levels and treatment strategy. Prognostic factors associated with survival outcomes were also explored. **Results**: A total of 65 patients were included, of whom 36 received pembrolizumab plus chemotherapy and 29 received pembrolizumab monotherapy. The estimated median PFS and OS for the entire cohort were 24.2 months (95% CI, 6.5–33.0) and 34.6 months (95% CI, 6.5–62.7), respectively. Patients with very high PD-L1 expression (TPS ≥ 90%) experienced significantly longer survival outcomes compared with those with a TPS of 50–89%, and a PD-L1 TPS ≥ 90% remained an independent prognostic factor for OS. When treatment strategies were compared across the entire cohort, no statistically significant differences in PFS or OS were observed between immunotherapy monotherapy and chemo-immunotherapy. Hypertension was identified as an independent negative prognostic factor for PFS. In patients with a PD-L1 TPS ≥ 90%, survival outcomes numerically favored pembrolizumab monotherapy, although this difference did not reach statistical significance. **Conclusions**: In this real-world cohort of patients with PD-L1 high metastatic NSCLC, PD-L1 expression, particularly very high TPS levels, was strongly associated with survival outcomes. While no survival differences were observed between treatment strategies in the overall population, pembrolizumab monotherapy may represent an appropriate first-line option in selected patients with a PD-L1 TPS ≥ 90%. Larger prospective studies are warranted to refine treatment selection in this setting.

## 1. Introduction

In 2022, lung cancer was the most commonly diagnosed malignancy and the leading cause of cancer-related mortality worldwide. Despite a recent shift in incidence ranking, it remains the primary contributor to global cancer mortality [1,2]. Non-small cell lung cancer (NSCLC) accounts for approximately 85% of all lung cancer cases [3]. For decades, platinum-based chemotherapy represented the only available first-line treatment for patients with metastatic NSCLC [4]. The introduction of immune checkpoint inhibitors (ICIs) marked a major paradigm shift in the treatment of advanced NSCLC. Programmed death-ligand 1 (PD-L1) expression was initially identified as a key predictive biomarker for response to immune checkpoint inhibition in the second-line setting, with higher expression levels consistently associated with greater clinical benefit from immunotherapy [5]. Building upon this biomarker-driven evidence, immune checkpoint inhibitors (ICIs) subsequently demonstrated meaningful clinical activity in the first-line setting from 2016 onward [6]. In patients with high PD-L1 expression (≥50%), pembrolizumab, atezolizumab, and cemiplimab demonstrated superior survival outcomes compared with chemotherapy in pivotal randomized trials, leading to their widespread adoption as standard first-line treatment options in clinical practice [6,7,8]. Despite the demonstrated efficacy of ICIs as monotherapy in this patient population, chemo-immunotherapy combinations with established clinical benefit remain widely used in routine practice [9,10]. In clinical decision-making, combination therapy is frequently considered for patients requiring rapid disease control, never-smokers, or those with tumors harboring molecular alterations associated with primary immunotherapy resistance, such as STK11 or KEAP1 mutations, given the relatively lower efficacy of immunotherapy alone in these subgroups; however, there is currently no consensus on the optimal selection between chemo-immunotherapy and immunotherapy alone in the first-line setting [11,12,13,14]. Accordingly, in a real-world cohort of patients with metastatic NSCLC and a PD-L1 TPS ≥ 50% treated at our center, we aimed to evaluate clinical and molecular factors associated with the choice and outcomes of first-line chemo-immunotherapy versus immunotherapy alone, with the goal of identifying patient subgroups that may benefit preferentially from each treatment strategy.

## 2. Methods

### 2.1. Study Design and Participants

Patients with metastatic NSCLC who initiated first-line treatment at the Istanbul Medipol University, Department of Medical Oncology between July 2017 and December 2024 were retrospectively reviewed. Eligible patients had a PD-L1 tumor proportion score (TPS) ≥ 50% and received either pembrolizumab monotherapy or pembrolizumab in combination with chemotherapy as first-line treatment. Tumor staging was performed according to the 8th edition of the American Joint Committee on Cancer (AJCC) and the Union for International Cancer Control (UICC) staging system, based on an integrated evaluation of clinical and radiological findings. A total of 65 patients who met the following inclusion criteria were enrolled in the study: age ≥ 18 years; histologically confirmed metastatic NSCLC; absence of actionable driver mutations; received pembrolizumab monotherapy or pembrolizumab plus chemotherapy as first-line treatment; and an Eastern Cooperative Oncology Group (ECOG) performance status (PS) of 0–2 [15].

### 2.2. Treatment Protocols

Patients who received chemo-immunotherapy were treated with carboplatin AUC 5 (area under the curve) in combination with pembrolizumab (200 mg) and either paclitaxel (175 mg/m^2^ intravenously) or pemetrexed (500 mg/m^2^ intravenously), with pemetrexed administered only in patients with non-squamous histology, on a 21-day cycle. After four or six cycles of combination therapy, patients without disease progression continued maintenance treatment with pembrolizumab (200 mg every 21 days, intravenously), with or without pemetrexed (500 mg/m^2^, intravenously) according to histology and clinical discretion. Patients treated with pembrolizumab monotherapy received pembrolizumab 200 mg every 21 days intravenously from treatment initiation until disease progression or for 2 years (35 cycles of pembrolizumab).

### 2.3. Data Collection

The following clinical and pathological data were extracted from medical records: sex, age at diagnosis, smoking status, disease stage at diagnosis, ECOG PS, sites of metastasis, histological subtype (adenocarcinoma or squamous cell carcinoma), chemotherapy regimen (carboplatin plus paclitaxel or carboplatin plus pemetrexed), and PD-L1 tumor proportion score (TPS).

### 2.4. Efficacy

The primary endpoints of the study were progression-free survival (PFS) and overall survival (OS). PFS was defined as the time from initiation of first-line treatment to documented disease progression, death from any cause, or the most recent follow-up, whichever occurred first. OS was calculated from the date of treatment initiation to death from any cause, with patients alive at last follow-up censored at that time point. Tumor responses were evaluated using the Response Evaluation Criteria in Solid Tumors (RECIST), version 1.1 [16]. Treatment responses were categorized as complete response (CR), partial response (PR), stable disease (SD), or progressive disease (PD). ORR was defined as the percentage of patients who achieved either CR or PR as their best overall response, while the disease control rate (DCR) encompassed patients who achieved CR, PR, or SD [16].

### 2.5. Safety

Treatment-related adverse events (TRAEs) were graded according to the Common Terminology Criteria for Adverse Events (CTCAE), version 5.0, established by the National Cancer Institute. The occurrence of TRAEs was documented separately for grade 1–2 (mild to moderate) and grade 3–4 (severe to potentially life-threatening) events [17]. For analytical purposes, TRAEs of special interest included fatigue, nausea, vomiting, constipation, diarrhea, loss of appetite, anemia, neutropenia, and thrombocytopenia. Additionally, immune-related adverse events of interest included hypothyroidism, hypophysitis, adrenal insufficiency, pneumonitis, hepatitis, nephritis, colitis, arthritis and myocarditis. The percentage of patients who experienced each TRAE was recorded for the entire study cohort.

### 2.6. Statistical Analysis

All data obtained from the study cohort were pooled for statistical analysis. Baseline characteristics were summarized using descriptive statistics, including counts and percentages for categorical variables, and means with standard deviations or medians with ranges for continuous variables, as appropriate. Survival analyses were performed using the Kaplan–Meier method, with comparisons between treatment groups conducted using the log-rank test. To explore factors associated with PFS and OS, cox proportional hazards regression analyses were applied. Variables demonstrating statistical significance in univariate analyses were subsequently incorporated into multivariate models using a stepwise selection method. Factors associated with treatment response were additionally examined through binary logistic regression analysis. Effect estimates were reported as hazard ratios (HRs) along with their corresponding 95% confidence intervals (CIs). All statistical analyses were conducted using IBM SPSS Statistics software (version 27.0; IBM Corp., Armonk, NY, USA). A two-sided *p* value < 0.05 was considered statistically significant.

## 3. Results

### 3.1. Patient Characteristics

Of the 65 patients included in the study, 52 were male (80.0%), and the median age at diagnosis was 69 years (range, 43–87). Most patients had an ECOG PS of 0 (n = 47, 72.3%). The majority of patients were either current or former smokers (n = 55, 84.6%). Most patients presented with metastatic disease at the time of diagnosis (n = 57, 87.7%). With respect to histology, adenocarcinoma was observed in 53 patients (81.5%), while 12 patients (18.5%) had squamous cell carcinoma. The most common metastatic sites were the bone (n = 27, 41.5%) and the brain (n = 23, 35.4%). Regarding PD-L1 expression, 39 patients (60.0%) had a PD-L1 TPS of 50–89%, whereas 26 patients (40.0%) had a TPS of ≥90%. Baseline patient characteristics are summarized in Table 1.

### 3.2. Survival Outcomes and Treatment Responses

The median PFS for the entire study cohort was 24.2 months (95% CI, 6.5–33.09). In univariate analysis, the presence of hypertension was identified as a negative prognostic factor for PFS (*p* = 0.017), whereas a PD-L1 TPS ≥ 90% was associated with a favorable prognostic impact (*p* = 0.037). In the multivariate analysis for PFS, the PD-L1 TPS did not retain statistical significance (*p* = 0.056), whereas the presence of hypertension remained an independent negative prognostic factor (*p* = 0.027). The results of the univariate and multivariate analyses of prognostic factors evaluated for PFS are summarized in Table 2. In the entire study population, the median OS was 34.6 months (95% CI, 6.5–62.99). In univariate analysis, both the presence of hypertension and the PD-L1 TPS were identified as significant prognostic factors for OS (*p* = 0.042 and *p* = 0.028, respectively). However, in the multivariate analysis, only the PD-L1 TPS was found to be an independent prognostic factor (*p* = 0.024). The results of the univariate and multivariate analyses of prognostic factors for OS are listed in Table 3. The median number of immunotherapy cycles administered was 15 (range: 2–35). Among the 65 patients, 10 (15.4%) achieved a CR, 40 achieved a PR (61.5%), 3 had SD (4.6%) and 12 experienced PD (18.5%). The ORR was calculated as 76.9%, and the DCR as 81.5%.

### 3.3. Survival Outcomes According to PD-L1 Expression and Treatment Regimen

**Figure 1:**
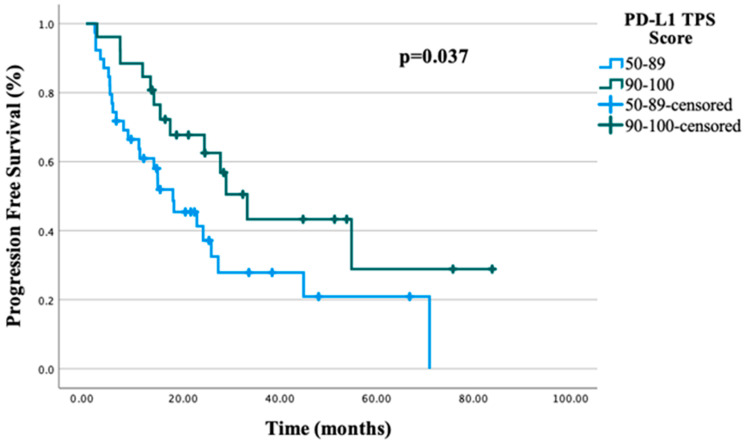
PFS according to PD-L1 TPS.

**Figure 2:**
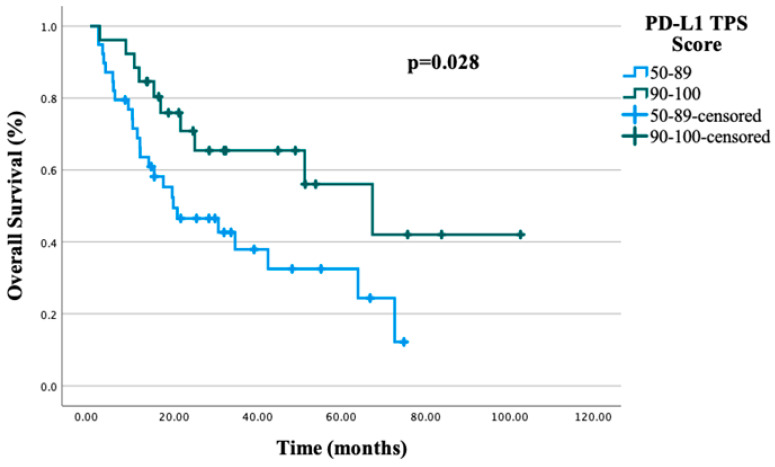
OS according to PD-L1 TPS.

**Figure 3:**
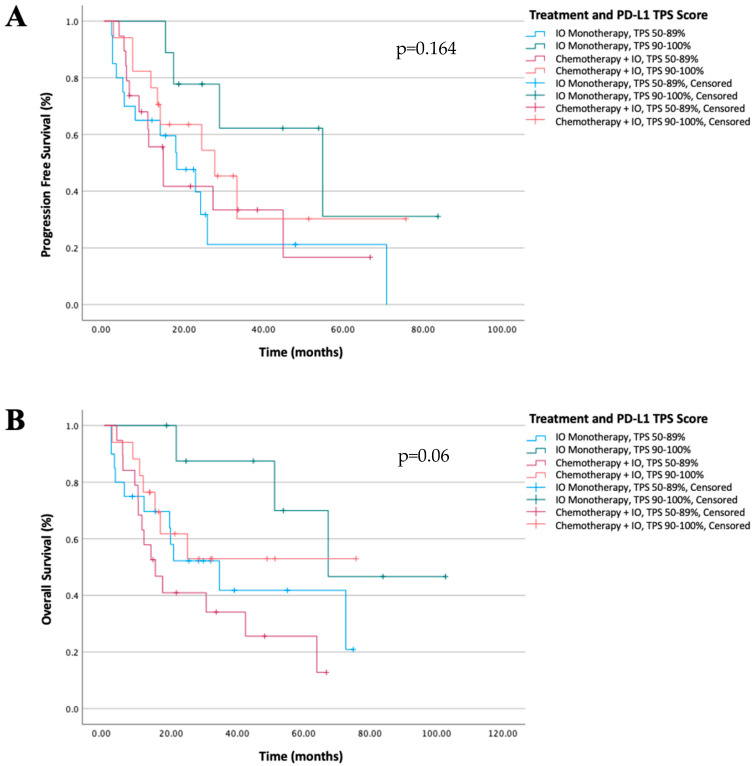
Survival according to treatment option and PD-L1 TPS: (**A**) PFS; (**B**) OS.

Of the 65 patients included in the study, 39 patients (60.0%) had a PD-L1 TPS of 50–89%, while 26 patients (40.0%) had a TPS ≥ 90%. Among patients with a PD-L1 TPS of 50–89%, 20 patients received immunotherapy monotherapy, whereas 19 patients were treated with chemo-immunotherapy. In the subgroup with a PD-L1 TPS ≥ 90%, 9 patients received immunotherapy monotherapy, while 17 patients were treated with chemo-immunotherapy. In the entire study population, patients with a PD-L1 TPS ≥ 90% demonstrated significantly longer PFS and OS in univariate analysis compared with those with a TPS of 50–89% (median PFS: 33.3 vs. 17.9 months, *p* = 0.037, and median OS: 67.3 vs. 19.8 months, *p* = 0.028, respectively). PFS and OS stratified by the PD-L1 TPS are illustrated in Figure 1 and Figure 2. When patients were further stratified according to the PD-L1 TPS and treatment regimen, no statistically significant differences in PFS or OS were observed between treatment groups. The PFS and OS trajectories according to the PD-L1 TPS and treatment strategy are shown in Figure 3.

### 3.4. Treatment-Related Adverse Events

The majority of reported treatment-related adverse events (TRAEs) were grade 1–2. Among grade 1–2 TRAEs, the most frequently observed events were fatigue (26.2%), anemia (24.6%), nausea (21.5%), constipation (18.5%), and decreased appetite (16.9%). Hematologic toxicities such as neutropenia (15.4%) and thrombocytopenia (10.8%) were also observed. The most common grade ≥ 3 TRAEs were nausea (6.2%), fatigue (4.6%), anemia (3.1%), neutropenia (3.1%), and vomiting (3.1%). Immune-related adverse events were observed in 27 patients (41.5%), with most being grade 1–2. The most frequently encountered immune-related adverse event was hypothyroidism (13.8%), followed by pneumonitis (13.9% overall; 7.7% grade 1–2 and 6.2% grade ≥ 3). Table 4 summarizes the TRAEs observed in the study cohort.

## 4. Discussion

In our study, the estimated median PFS and OS were 24.2 and 34.6 months, respectively. These survival outcomes appear to be notably longer than those reported in the existing literature [18,19]. We believe that several factors may have contributed to this finding. First, the baseline characteristics of our cohort were relatively favorable compared with those reported in previous studies. In particular, a large proportion of patients had an ECOG performance status of 0 (72.3%), which is higher than that reported in many pivotal trials and may have contributed to the improved survival outcomes observed in our cohort [13]. Second, among patients treated with chemo-immunotherapy, the proportion of patients who completed the full 35 cycles of pembrolizumab was 25% (n = 9) in our cohort, which is higher than the rates reported in pivotal clinical trials, where completion rates of approximately 13.9% have been described [19]. In contrast, the proportion of patients completing 35 cycles in the pembrolizumab monotherapy group was comparable to that reported in the literature (25.8% vs. 24.1%) [18]. A third contributing factor may be the durability of response observed in patients who completed 35 cycles of pembrolizumab. Of these 16 patients, 10 remained progression-free at the time of analysis, and 5 patients achieved OS exceeding five years, suggesting a sustained long-term benefit in a subset of patients. Fourth, the use of local ablative therapies in cases of oligoprogression, allowing continuation of the ongoing systemic treatment, may have further contributed to the prolonged survival outcomes observed in our cohort.

Consistent with observations reported in some previous studies, patients with baseline hypertension in our cohort experienced shorter PFS and OS compared with those without hypertension (median PFS: 14.0 vs. 27.7 months; median OS: 15.3 vs. 51.2 months) [20]. In univariate analysis, hypertension was identified as a significant prognostic factor for both PFS and OS. However, in the multivariate analysis, hypertension retained its prognostic significance only for PFS (*p* = 0.027; hazard ratio [HR], 0.475; 95% confidence interval [CI], 0.246–0.917). Given the relatively small sample size of the cohort and the predominance of male patients in the study population, this finding should be interpreted with caution.

In our study, patients with a PD-L1 TPS ≥ 90% demonstrated longer PFS compared with those with a TPS of 50–89%; however, this association did not retain statistical significance in the multivariate analysis. In contrast, while a PD-L1 TPS ≥ 90% was associated with prolonged OS in the univariate analysis, this relationship remained statistically significant in the multivariate model, indicating that high PD-L1 expression (≥90%) represents an independent prognostic factor for OS. These findings are consistent with previously published data in which patients with very high PD-L1 expression (TPS ≥ 90%) have been shown to experience longer PFS and OS compared with those with a PD-L1 TPS of 50–89% [21]. Our results therefore further support the prognostic relevance of very high PD-L1 expression in patients with metastatic NSCLC.

In this study, 36 patients received pembrolizumab plus chemotherapy, whereas 29 patients were treated with pembrolizumab monotherapy as first-line treatment. No statistically significant differences were observed between the two treatment groups in terms of PFS or OS. These findings are consistent with previously published data in which no significant differences in OS have been reported between immunotherapy monotherapy and chemo-immunotherapy among patients with a PD-L1 TPS ≥ 50%, despite some reported differences in PFS [14,22,23,24].

Evaluation of the Kaplan–Meier survival curves revealed that, despite the lack of statistical significance, patients with a PD-L1 TPS ≥ 90% who received pembrolizumab monotherapy appeared to have more favorable PFS and OS patterns. In patients with very high PD-L1 expression (≥90%), the incremental benefit of adding chemotherapy to immunotherapy has previously been reported to be limited, a finding supported by the existing literature [24,25]. In this context, while several studies have demonstrated numerically longer OS with chemo-immunotherapy compared with immunotherapy alone, these differences have generally not reached statistical significance. In contrast, in our study, the median PFS and OS were numerically longer in the immunotherapy monotherapy group among patients with very high PD-L1 expression (54.83 months vs. 27.76 months for PFS and 67.36 months vs. NR for OS) [22].

In our study cohort, although PD-L1 expression appeared to provide insight into treatment selection, no significant association was observed between treatment outcomes and other clinical factors that have previously been suggested to favor immunotherapy monotherapy, such as male sex and a history of smoking [14]. This lack of association is most likely attributable to the limited sample size of our study rather than the absence of a true biological effect.

Several limitations of this study should be considered when interpreting the results. First, the retrospective nature of the analysis inherently carries the risk of selection bias and unmeasured confounding, which may have affected the observed associations. In addition, treatment allocation between pembrolizumab monotherapy and chemo-immunotherapy was determined by the treating physician according to routine clinical practice, taking into account factors such as disease burden, risk of rapid disease progression, smoking history, and patients’ ECOG performance status, which may have introduced potential selection bias between the treatment groups. Second, as the study was conducted at a single institution, the generalizability of the findings to broader patient populations may be limited. In addition, a further limitation of our study is that comprehensive molecular profiling was not performed in all patients, which may have limited our ability to evaluate the impact of specific genetic alterations on treatment outcomes. Finally, and perhaps most importantly, the relatively small sample size, particularly within subgroup analyses, may have reduced the statistical power to detect meaningful differences between treatment strategies. Therefore, the subgroup findings should be interpreted with caution.

## 5. Conclusions

In conclusion, PD-L1 TPS appears to be an important determinant in treatment decision-making for patients with metastatic NSCLC. In our cohort, patients with very high PD-L1 expression (TPS ≥ 90%) showed numerically longer survival outcomes; however, these findings did not reach statistical significance and should therefore be interpreted with caution. Additional clinical characteristics, such as sex and smoking history, may also contribute to treatment selection, although these factors were not statistically significant in our study. Larger studies with extended follow-up and larger patient populations are needed to further clarify how clinical and pathological characteristics may help guide optimal treatment strategies in this setting.

## Figures and Tables

**Table 1:** General characteristics of 65 patients.

Characteristic	n (%)
Age	Median: 69 (range, 43–87)
Sex	
Male	52 (80)
Female	13 (20)
ECOG performance status	
0	47 (72.3)
1	16 (24.6)
2	2 (3.1)
Smoking history	
Never smoked	10 (15.4)
Ex-smoker	39 (60)
Current smoker	16 (24.6)
Hypertension	
Present	23 (35.4)
Absent	42 (64.6)
Diabetes	
Present	15 (23.1)
Absent	50 (76.9)
Tumor Histology	
Adenocarcinoma	53 (81.5)
Squamous cell carcinoma	12 (18.5)
Disease status at first diagnosis	
De novo metastatic	57 (87.7)
Recurrent disease	8 (12.3)
Site of metastasis	
Bone	27 (41.5)
Brain	23 (35.4)
Adrenal	19 (29.2)
Distant lymph node	15 (23.1)
Contralateral lung	9 (13.8)
Liver	7 (10.8)
PD-L1 TPS	
50–89%	39 (60)
≥90%	26 (40)
Treatment	
Pembrolizumab monotherapy	29 (44.6)
Chemotherapy + pembrolizumab	36 (55.4)

Abbreviations: ECOG, Eastern Cooperative Oncology Group; PD-L1, Programmed Death-Ligand 1; TPS: Tumor Proportion Score.

**Table 2:** Univariate and multivariate analyses for progression-free survival.

Features	n (%)	Median PFS (Months)	Univariate *p* Value	Multivariate *p* Value	HR (CI%)
Sex			0.43		
Male	52 (80)	22.9			
Female	13 (20)	27.6			
Smoking history			0.91		
Never smoked	10 (5.4)	25.8			
Ex-smoker	39 (60)	24.2			
Current smoker	16 (24.6)	28.9			
Hypertension			0.017	0.027	0.475 (0.246–0.917)
Present	23 (35.4)	14			
Absent	42 (64.6)	27.7			
Diabetes mellitus			0.47		
Present	15 (23.1)	17.4			
Absent	50 (76.9)	24.4			
Tumor histology			0.49		
Adenocarcinoma	53 (81.5)	24.2			
Squamous cell carcinoma	12 (18.5)	NR			
Site of metastasis					
Bone	27 (41.5)	17.4	0.438		
Brain	23 (35.4)	27.3	0.484		
Adrenal	19 (29.2)	24.2	0.218		
Distant lymph node	15 (23.1)	27.7	0.888		
Contralateral lung	9 (13.8)	24.5	0.676		
Liver	7 (10.8)	7.7	0.241		
Initial disease stage			0.656		
De novo metastatic	57 (87.7)	24.2			
Recurrent disease	8 (12.3)	28.93			
Treatment			0.85		
Pembrolizumab + chemotherapy	36 (55.4)	24.46			
Pembrolizumab	29 (44.6)	24.2			
PD-L1 TPS			0.037	0.056	0.513 (0.258–1.018)
50–89%	39 (49.2)	17.96			
≥90%	26 (50.8)	33.3			

Abbreviations: PD-L1, Programmed Death-Ligand 1; TPS: Tumor Proportion Score.

**Table 3:** Univariate and multivariate analyses for overall survival.

Features	n (%)	Median OS (Months)	Univariate *p* Value	Multivariate *p* Value	HR (CI%)
Sex			0.221		
Male	52 (80)	30.6			
Female	13 (20)	NR			
Smoking history			0.908		
Never smoked	10 (5.4)	21.6			
Ex-smoker	39 (60)	42.4			
Current smoker	16 (24.6)	30.6			
Hypertension			0.042	0.052	0.498 (0.246–1)
Present	23 (35.4)	15.3			
Absent	42 (64.6)	51.2			
Diabetes mellitus			0.693		
Present	15 (23.1)	30.6			
Absent	50 (76.9)	42.4			
Tumor histology			0.771		
Adenocarcinoma	53 (81.5)	42.4			
Squamous cell carcinoma	12 (18.5)	34.6			
Site of metastasis					
Bone	27 (41.5)	20.8	0.261		
Brain	23 (35.4)	42.4	0.68		
Adrenal	19 (29.2)	72.6	0.438		
Distant lymph node	15 (23.1)	63.9	0.38		
Contralateral lung	9 (13.8)	34.6	0.594		
Liver	7 (10.8)	19.6	0.43		
Initial disease stage			0.449		
De novo metastatic	57 (87.7)	30.6			
Recurrent disease	8 (12.3)	51.2			
Treatment			0.165		
Pembrolizumab + chemotherapy	36 (55.4)	24.96			
Pembrolizumab	29 (44.6)	67.36			
PD-L1 TPS			0.028	0.024	0.415 (0.193–0.889)
50–89%	39 (49.2)	19.86			
≥90%	26 (50.8)	67.36			

Abbreviations: PD-L1, Programmed Death-Ligand 1; TPS: Tumor Proportion Score.

**Table 4:** Treatment-related adverse events.

Adverse Event	Grade 1 or 2 n (%)	Grade 3 or 4 n (%)
All adverse events	22 (33.8%)	19 (29.2%)
Fatigue	17 (26.2%)	3 (4.6%)
Anemia	16 (24.6%)	2 (3.1%)
Nausea	14 (21.5%)	4 (6.2%)
Constipation	12 (18.5%)	1 (1.5%)
Decreased appetite	11 (16.9%)	1 (1.5%)
Neutropenia	10 (15.4%)	2 (3.1%)
Vomiting	10 (15.4%)	2 (3.1%)
Thrombocytopenia	7 (10.8%)	0
Diarrhea	3 (4.6%)	1 (1.5%)
Any immune-related adverse events	19 (29.2%)	8 (12.3%)
Hypothyroidism	9 (13.8%)	0
Pneumonitis	5 (7.7%)	4 (6.2%)
Adrenal insufficiency	3 (4.6%)	0
Hypophysitis	2 (3.1%)	0
Arthritis	2 (3.1%)	0
Hyperthyroidism	1 (1.5%)	0
Hepatitis	1 (1.5%)	1 (1.5%)
Nephritis	1 (1.5%)	1 (1.5%)
Colitis	1 (1.5%)	1 (1.5%)
Myocarditis	0	1 (1.5%)

## Data Availability

Due to the sensitive nature of the data, it is not publicly accessible; however, it may be provided by the corresponding author upon reasonable request.
